# Correction to “Salvage Trans‐Sylvian Peri‐Insular Hemispherotomy After Embolic Hemispherectomy”

**DOI:** 10.1002/cns3.70072

**Published:** 2026-05-11

Baumgartner, M.E., Kessler, S., Galligan, K., Baumgartner, J.E. and Kennedy, B.C. (2025), Salvage Trans‐Sylvian Peri‐Insular Hemispherotomy After Embolic Hemispherectomy. Annals of the Child Neurology Society, 3: 110‐114. https://doi.org/10.1002/cns3.70003.

“A Case Report and Review of the Literature” is added as the article's subtitle.

We apologize for this error.

